# Airway management in children with hemifacial microsomia: a restropective study of 311 cases

**DOI:** 10.1186/s12871-020-01038-2

**Published:** 2020-05-20

**Authors:** Jin Xu, Xiaoming Deng, Fuxia Yan

**Affiliations:** 1 Department of Anesthesiology, Plastic Surgery Hospital, Chinese Academy of Medical Sciences and Peking Union Medical College, No. 33, Ba Da Chu Road, Shi Jing Shan, Beijing, 100144 China; 2 Department of Anesthesiology, Fuwai Hospital, National Center for Cardiovascular Diseases, Chinese Academy of Medical Sciences and Peking Union Medical College, 167 North Lishi Road, XiCheng District, Beijing, 100037 China

**Keywords:** Difficult, Airway, Children, Anesthesia, Hemifacial microsomia

## Abstract

**Background:**

Hemifacial microsomia (HFM) is a congenital craniofacial malformation which is associated with difficult airway. Anesthesiologists may experience difficult intubation in children with HFM. Mandibular distraction could increase the length of the mandible. Theoretically, it should be advantageous to laryngeal view during tracheal intubation. This study reviewed airway management in children with HFM, assessed the efficiency of direct laryngoscopy versus airway-visualizing equipment during the tracheal intubation and determined whether mandibular distraction could improve the laryngoscopic view in children with HFM.

**Methods:**

A retrospective review of cases involving children with HFM aged 5 to 17 years old underwent anesthesia from December 2016 to April 2019 at a single center was performed. The demographic data, preoperative airway assessments, procedure type, anesthetic technique, method of airway management, anesthetists’ comments on mask ventilation, laryngoscopy and intubation parameters were collected.

**Results:**

At last, 136 HFM children entered this study, a total of 311 anesthesia procedures were completed during the study period. Face mask ventilation was possible for most of children except 1 child (bilateral involvement) required two practitioners. The success rates of intubation for the primary video laryngoscopy and fibroscopy were both 100%, but 79.5% for direct laryngoscopy (*P* < 0.001). 95 (38.9%) children who had difficult laryngoscopic view (DLV) were significantly correlated with failed direct laryngoscopy (*P* < 0.001). Airway-visualizing equipment (video laryngoscope and Fiberscope) was the primary airway technique in 3 (75%) bilaterally involved children. 60 children underwent both mandibular distraction osteogenesis and the removal of distractor. The laryngoscopic views improved in 26 (43%) children after treatment with mandibular distraction (*P* < 0.001).

**Conclusions:**

Airway-visualizing equipment can be effectively utilized for intubation in HFM children with DLV. Mandibular distraction could improve the laryngeal view effectively.

## Background

Hemifacial microsomia (HFM) is a congenital craniofacial malformation that features hypoplasia and asymmetry in skeletal tissue as well as in soft tissue [1]. As HFM involves the structure of the first and second pharyngeal arches, it presents across a wide area, which includes the maxilla, mandible, external ear, middle ear ossicles, facial and trigeminal nerves, temporal bone, and muscles of facial expression [2].

As the second most common facial birth defect after cleft and palate deformities, HFM has an estimated incidence ranging from 1:3500 to 1:5600 [3]. Anesthesiologists who anesthetize children are likely to encounter this disorder. Establishing airway management for patients with HFM is a challenge for anesthesiologists. Because HFM is associated with mandibular hypoplasia and temporomandibular joint abnormalities, these malformations can cause difficulties for direct laryngoscopy and endotracheal intubation.

After McCarthy et al. first reported lengthening the human mandible by gradual distraction, mandibular distraction osteogenesis has gradually become the preferred technique to treat HFM because this process allows for a stable expansion of the mandible with concurrent lengthening and expansion of the surrounding muscle and soft tissue [4]. Theoretically, the distraction device could improve mouth opening and alter the laryngoscopic view during tracheal intubation. The current literature on airway management in HFM patients is limited to case reports and very small case series. However, the efficacy of different airway techniques in pediatric airways remains unknown.

The primary objective of the study was to assess the efficiency of direct laryngoscope versus airway-visualizing equipment during the tracheal intubation for children with HFM. The second objective was to determine whether mandibular distraction could improve visualization of the laryngeal structure in HFM children with DLV.

## Methods

### Patients

Approval for the study was obtained from our institution’s Ethics.

Committee (Reference No. ZX2019–21, date of approval 23/5/2019). Data of all intubations for children with HFM (aged 5 to 17 years) performed at the Plastic Surgery Hospital, Chinese Academy of Medical Science, Peking Union Medical College, Beijing, China (Plastic Surgery Hospital, Chinese Academy of Medical Science, Peking Union Medical College) from December 2016 to April 2019 were retrospectively evaluated. The selection period was from the time of establishing an electronic medical records system and integrating the clinical data in our hospital to the present. The diagnosis of HFM was confirmed by the craniofacial surgery team.

### Materials

Using anesthesia records and a difficult airway database, the following data were collected: basic demographics of the patients; preoperative airway assessments (modified Mallampati classification (MMP), thyromental distance (TMD), interincisor gap (IIG), forward protrusion of the mandible (FPM)); reasons for anesthesia according to procedure type; anesthetic technique used; anesthetists’ comments on mask ventilation, laryngoscopy, and intubation; the first attempt airway device, rescue device(s) and number of attempts;

A four-point scale, originally described by Han et al. [5], was used to define and classify the face mask ventilation process, as follows:

- Grade I: ventilated by mask;
- Grade II: ventilated by mask with an oropharyngeal airway/adjuvant with or without a muscle relaxant;
- Grade III: difficult ventilation (inadequate, unstable, or requiring two practitioners) with or without a muscle relaxant;
- Grade IV: unable to mask ventilate with or without a muscle relaxant.

The recorded description of the direct laryngoscopic view was graded using the Cormack-Lehane (CL) classification [6], as follows:

- Grade I: full view of the glottis;
- Grade II: partial view of the glottis or arytenoids;
- Grade III: partial or full view of the epiglottis;
- Grade IV: no visualization of either the glottis or epiglottis.

Grades I and II were defined as easy laryngoscopic view (ELV), while grades III and IV were defined as DLV.

The MMP was used to evaluate the trachea with the aid of a light; the patient was sitting upright with the head in a neutral position, mouth fully opened and tongue fully protruded [7]. The patients were categorized as follows:

- Class I: soft palate, fauces, anterior and posterior tonsillar pillars, and the entire uvula are visible;
- Class II: soft palate, fauces, and uvula are visible;
- Class III: soft palate and base of uvula are visible;
- Class IV: only the hard palate is visible.

The TMD was measured with a rigid rule from the thyroid notch to the lower border of the mandibular mentum while the head was fully extended and the mouth was closed. Considering that TMD does not consider an individual’s body proportions, the ratio of height to TMD (RHTMD) [8] was calculated to predict DLV. The RHTMD was calculated as height (in cm)/TMD (in cm).

The IIG was measured between the upper and lower incisors at the midline when the mouth was fully opened.

FPM was assessed as the ability to protrude the mandibular incisors in front of the maxillary incisors and was graded as follows:

- Grade I: the mandibular incisors could protrude in front of the maxillary incisors;
- Grade II: the mandibular incisors could not protrude in front of the maxillary incisors.

All decisions and treatments regarding airway management were at the discretion of the anesthesia provider and were not influenced by the study design.

### Statistics

Statistical analyses were performed by using SPSS 23 for Windows (SPSS Inc., Chicago, IL, USA). The data are presented as frequencies (both absolute and percentage). A proportional analysis was performed via Fisher’s exact test. The McNemar test was used to test for significance in pass success of first attempt of direct laryngoscopy and DLV changes from mandibular distraction osteogenesis (First-stage) to removal of distractors (Second-stage). Spearman rank correlation was used to test for the relationship between the DLV and failed intubation by direct laryngoscopy.

Airway predictors were entered into a multivariate logistic regression analysis to determine the independent predictors for DLV. It is now believed that microtia is a forme fruste of HFM [2]. Estimating a 30% incidence of difficult intubation in school-aged children with microtia [9], a sample size of 141 patients was calculated to have a power of 0.85 and a significance level of 0.05 to detect a difference in predictors between the ELV and DLV groups with PASS software (version 11; NCSS LLC, Kaysville, UT, USA).

The ability of each independent predictor to predict DLV and the cutoff values for each predictor were evaluated by the receiver operating characteristic (ROC) curve analysis. The sensitivity, specificity, positive predictive value (PPV), negative predictive value (NPV) were calculated for significant variables identified from the ROC curves. Different combinations of predictors were also analyzed. ROC curve analysis was conducted with MedCalc Statistical Software 12.7.7 (MedCalc Software bvba, Ostend, Belgium; http://www.medcalc.org; 2013). *P* < 0.05 was considered statistically significant.

## Result

For the 136 children known to have HFM, a total of 311 general anesthesia procedures were completed during the study period. Of the 105 children who received general anesthesia on at least one occasion, 82 (62%) were males. 4 patients had bilateral involvement. The patients’ characteristics and anesthesia data are shown in Table 1. In total, 62% of the procedures involved the mandible, 32% involved the ear, and 6% involved other structures.

**Table 1:** The demographics and anesthesia date

Age(years) (*n* = 311)	
5–8	173(55.6%)
9–13	102(32.8%)
14–17	36(11.6%)
Sex (*n* = 136)	
Male	84(62%)
Female	52(38%)
Side involved (n = 311)	
Left	157(50.4%)
Right	150(48.2%)
Bilateral	4(1.3%)
Anesthesia (n = 311)	
Endotracheal tube	307(98.7%)
Intubation under spontaneous breathing	1(0.3%)
Intubation with muscle relaxants	306 (99.7%)
LMA	4(1.2%)
Mask ventilation classification (n = 311)	
I	292 (93.8%)
II	18 (5.7%)
III	1 (0.3%)
IV	0 (0%)

Mask ventilation classification, Class I: ventilated by mask; Class II: ventilated by mask with oral airway/adjuvant with or without muscle relaxant; Class III: difficult ventilation (inadequate, unstable, or requiring two providers) with or without muscle relaxant; Class IV: unable to mask ventilate with or without muscle relaxant

### Anesthesia technique

All anesthesia procedures were performed by specialist anesthetists. Inhalation induction was used for 7 procedures (2%) by sevoflurane, and intravenous induction was used for 304 procedures (98%) with 0.2–0.3 μg/kg of sufentanil, propofol 2–3 mg/kg intravenously. Muscle relaxants were administered before intubation and the use of the laryngeal mask airway for 310 procedures with 0.6 mg/kg rocuronium. Only 1 procedure in 1 child with bilateral involved was intubated under spontaneous breathing without muscle relaxants. The children’s head was placed in the ‘sniffing position’when performing intubation.

### Airway management

The anesthetists described the initial face mask ventilation process after administering anesthesia as grade I for 292 procedures (93.8%), grade II for 18 procedures (5.7%, requiring oropharyngeal airway) and grade III for 1 procedure (0.3%, requiring two practitioners). Grade III was found in the children with bilateralinvolvement who required two practitioners. For other anesthesia procedures, mask ventilation was possible.

**Fig. 1:**
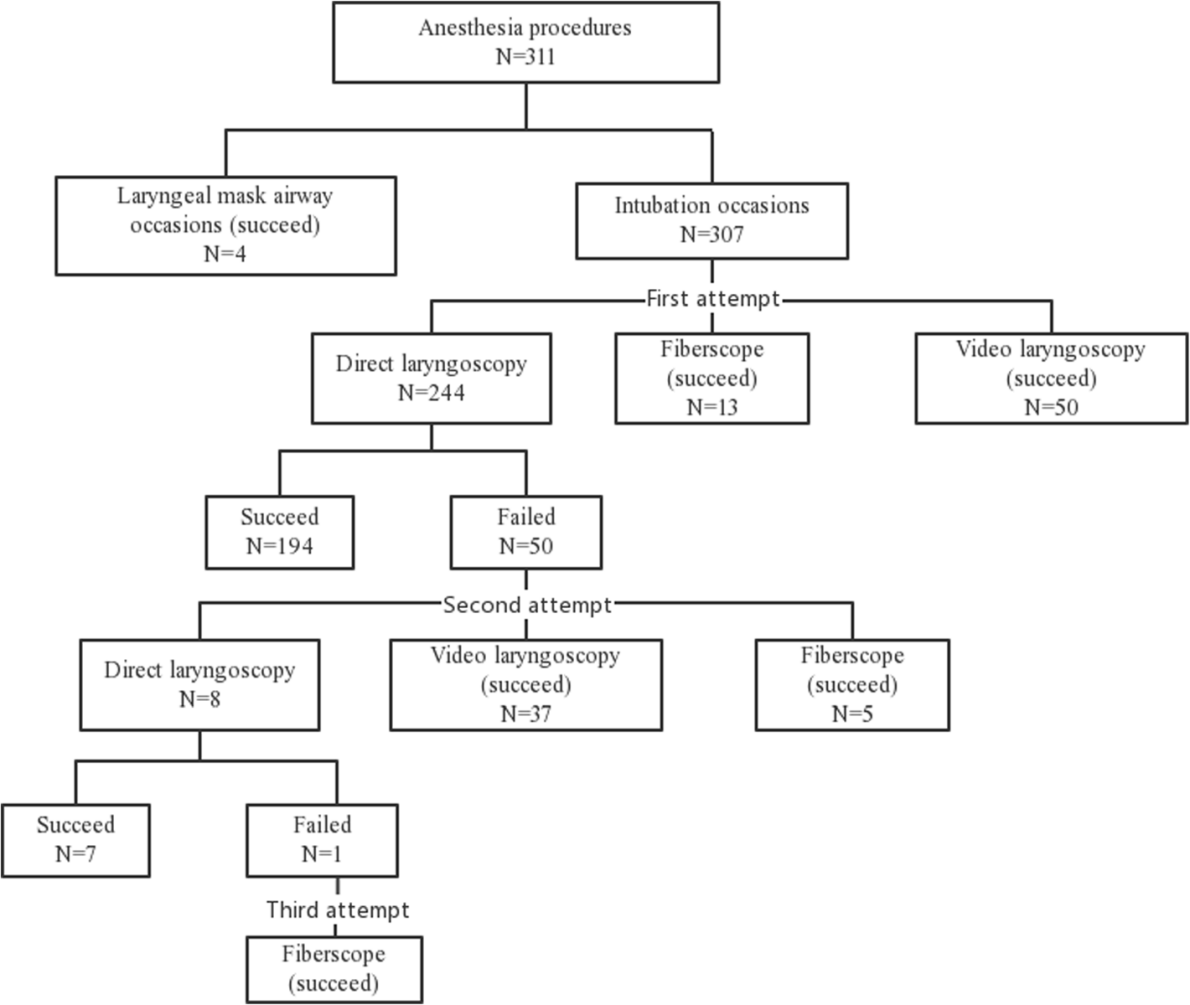
Flow diagram of anesthesia procedures

The methods of airway management for each anesthesia episode were as follows(Fig. 1): laryngeal mask airway was used for 4 anesthesia procedures in 4 children, and all ventilation procedures were successful; tracheal intubation was planned for 307 procedures in 132 children, with 244 procedures in 76 children undergoing direct laryngoscopy, 50 procedures in 44 children undergoing channeled video laryngoscopy(Tosight® TSEL-2000, China) and 13 procedures in 12 children undergoing fibroscopy. The first pass success rates of video laryngoscopy and fibroscopy were both 100%. However, direct laryngoscopy had a lower success rate (*n* = 194, 79.5%) (*P* < 0.001). In 50 cases of first failed intubation, 42 cases were converted to video laryngoscopy (*n* = 37, 74%) or fibroscopy (*n* = 5, 10%), and all procedures were successful. The other 8 cases (16%) underwent a second attempt of intubation by direct laryngoscopy, 7 cases were success, but 1 case failed the procedure twice, and the procedure was converted to fibroscopy, which was finally successful. 4 anesthesia procedures in 4 children were bilateral involvement. Among them, 2 procedures in 2 children were intubated with the assistance of a fiberscopy, 1 procedure underwent video laryngoscopy, 1 child with a grade IV laryngoscopic view underwent a blind intubation by direct laryngoscopy. A description of the laryngoscopic view was recorded for 238 (97.5%) of the 244 procedures intubated by directed laryngoscopy. A total of 95 (38.9%) procedures had CL grade 3 (*n* = 77) and grade 4 (*n* = 18) laryngeal views, which were significantly correlated with failed intubation (*P* < 0.001). Table 2 summarizes the method of intubation and CL classification.

**Table 2:** Summary of intubation devices (*n* = 307)

P	Direct laryngoscopy	Video laryngoscopy	Fiberscope	*P*-value
First attempt airway device(n = 307)	244	50	13	
First pass success(*n* = 257)	194(79.5%)	50(100%)	13(100%)	*P*<0.001*^*^
direct laryngoscopy view				
I	63(26.5%)			
II	80(33.6%)			
III	77(32.4%)			
IV	18(7.6%)			
Data missing	6			
Second pass success(*n* = 49)	7	37	5	
Third pass success(n = 1)			1	

^*^Fisher’s exact test; * Statistically significant difference (*P* < 0.05)

**Fig. 2:**
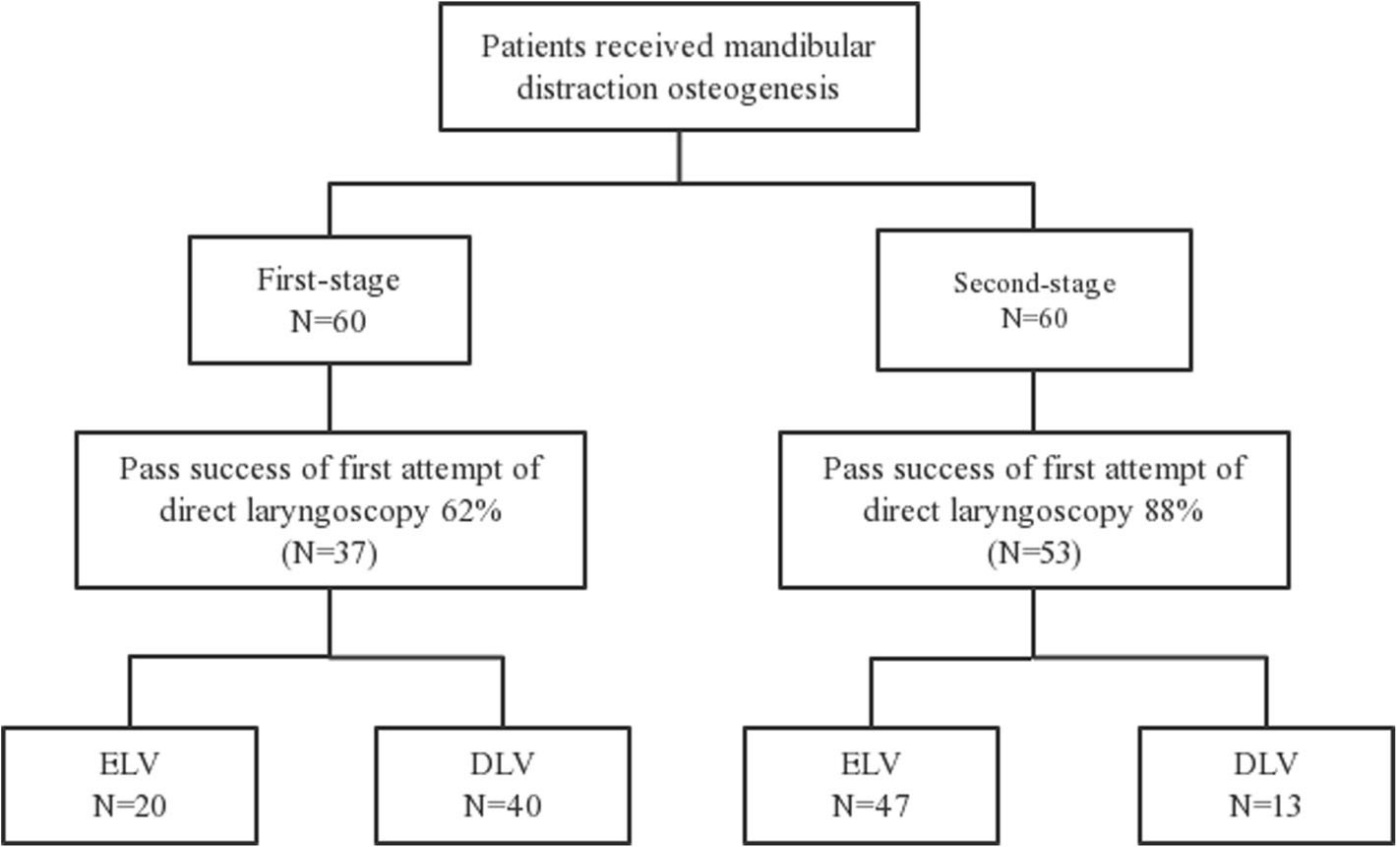
Laryngoscopic view of patients received mandibular distraction osteogenesis. ELV = Easy laryngoscopic view; DLV = Difficult laryngoscopic view

**Fig. 3:**
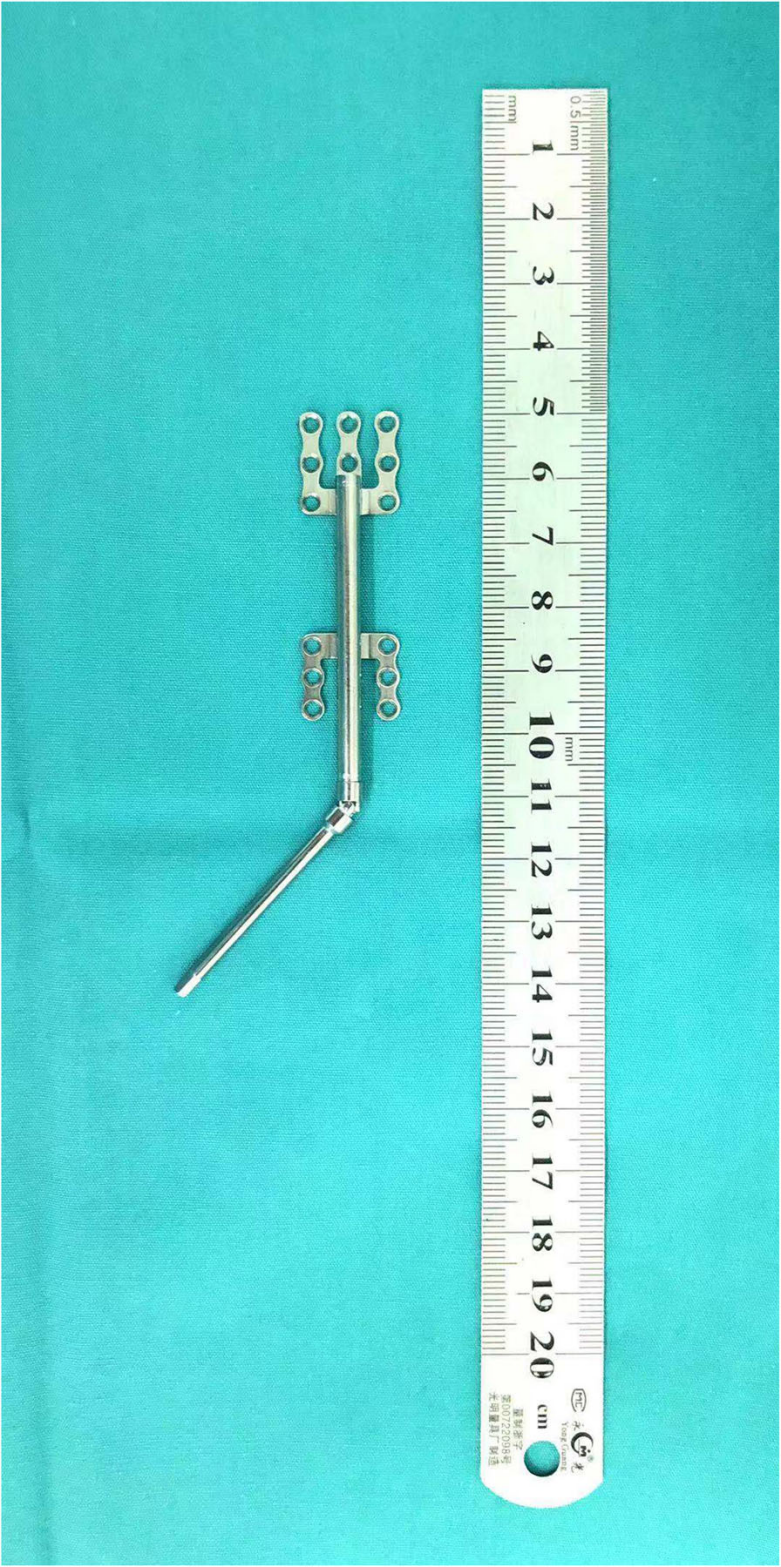
Mandibular distractor. A semiburied unidirectional distraction device (CIBEI MEDICAL INSTRUMENT CO, LTD, Ning Bo, China) . The distractor activation arm is pointing anteriorly

**Fig. 4:**
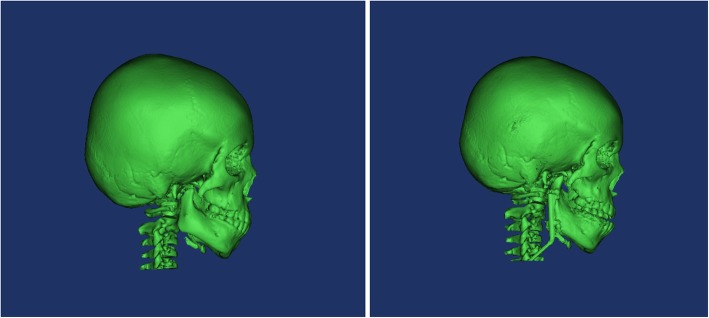
Preoperative three-digital image demonstrating mandibular distraction osteogenesis expand the mandible. a. Image collected before mandibular distraction osteogenesis. b. Image collected 6 months after mandibular distraction osteogenesis

A total of 60 children underwent both mandibular distraction osteogenesis(first-stage) and removal of distractors(second-stage) (Fig. 2). The distraction (Fig. 3) was started at a rate of 1 mm/day 4 to 7 days after the first-stage operation. The overall distraction distances ranged from 20 to 40 mm. And then the distractors were left in place for 4 to 10 months for consolidation. At last, the distractor was carried out to remove at the second-stage operation (Fig. 4). The laryngoscopic views and first attempt of intubation under direct laryngoscopy during first-stage and second-stage are presented in Table 3. Of these 60 children, only 1 child’s laryngoscopic view more difficult during second-stage after the procedures than first-stage undergoing direct laryngoscopy. A total of 26 (43%) children who had DLVs during the first-stage converted to ELVs during the second stage (*P* < 0.001). In total, 16 (27%) children, who failed intubation of the first attempt during the first stage, had a successful direct laryngoscopy after the procedures (*P* < 0.001).

**Table 3:** Laryngoscopic view of patients undergoing mandibular distraction osteogenesis(First-stage I) and removal of distractors(Second-stage)

PProcedure		First-stage (n = 60)	Second-stage (n = 60)	*P*-value
First attempt of direct laryngoscopy(*n* = 60)		60	60	
Pass success		37(62%)	53(88%)	*P*<0.001^*^
Laryngoscopic view				
ELV	I	3	20	*P*<0.001^*^
	II	17	27	
DLV	III	32	11	
	IV	8	2	

^*^McNemar test; *Statistically significant difference (*P* < 0.05)

*ELV* Easy laryngoscopic view, *DLV* Difficult laryngoscopic view

There were no cases of ‘can’t intubate, can’t ventilate’ situations, and no emergency cricothyroidotomy or tracheostomy airway management procedures were needed.

### Airway prediction

For 238 patients intubated by direct laryngoscopy, there was sufficient information about the airway assessments to predict difficult laryngoscopy. A multivariate logistic regression analysis showed that IIG, RHTMD and FPM were independent predictors of DLV (Table 4).

**Table 4:** Multivariate logistic regression showing the independent predictors of DLV(*n* = 238)

Predictors	β	Wald	P-value	OR	95% C.I.for OR	
					Lower	Upper
MMP(≥III)	0.299	0.553	0.457	1.349	0.613	2.967
IIG	−0.707	4.235	0.040*	0.493	0.252	0.967
RHTMD	0.128	15.766	< 0.001*	1.137	1.067	1.211
FPM	−1.450	9.065	0.003*	0.235	0.091	0.603

*Statistically significant difference (*P* < 0.05)

*DLV* Difficult laryngoscopic view, *MMP* Modified Mallampati classification, *IIG* Interincisor gap, *RHTMD* Ratio of height to thyromental distance, *FPM* Forward protrusion of the mandible

The ROC analysis to predict DLV (Table 5) revealed that RHTMD was the best predictor, with an AUC of 0.720, a sensitivity of 86.25%, and a specificity of 45.71%. The combination of RHTMD, FPM, and IIG was the best predictor for DLV, with an AUC of 0.782, a sensitivity of 94.12%, and a specificity of 88.57%.

**Table 5:** Statistical results of the predictors for predicting DLV

	AUC	95%CI	cut-off	Sensitivity %	Specificity%	PPV %	NPV %
IIG	0.625	0.543–0.702	≤3.5	55.41	68.35	62.1	62.1
FPM	0.609	0.528–0.687	>1	32.00	89.87	75.0	58.2
RHTMD	0.720	0.650–0.784	>28.83	86.25	45.71	54.8	81.4
RHTMD+FPM	0.755	0.678–0.822	> 0.6211	55.71	85.90	78.0	68.4
RHTMD+IIG	0.741	0.662–0.809	> 0.4073	74.29	62.82	64.2	73.1
IIG + FPM	0.695	0.615–0.767	> 0.3406	70.27	64.56	65.0	69.9
RHTMD+IIG + FPM	0.782	0.707–0.846	> 0.2857	94.12	88.57	62.6	83.7

*Statistically significant difference (*P* < 0.05)

*DLV* Difficult laryngoscopic view, *MMP* Modified Mallampati classification, *IIG* Interincisor gap, *RHTMD* Ratio of height to thyromental distance, *FPM* Forward protrusion of the mandible

## Discussion

This review of 311 anesthesia procedures of airway management in 136 children with HFM is the largest reported up to now. Our results provide evidence for the effective use of intubation equipment in children with HFM. Although video laryngoscopy and fibroscopy could improve the intubation success rate, direct laryngoscopy is still the first choice and standard technique for tracheal intubation in most cases. Children who presented difficult laryngeal visualization were correlated with failed intubation by direct laryngoscopy.

In the past decade, airway management techniques have changed dramatically. With the development of airway-visualizing devices that are increasingly used today, the selection of the primary airway device has shifted away from conventional direct laryngoscopy to manage difficult intubation. Owing to its portability and easy handling, video laryngoscopes may provide an accessible alternative to fibroscopy. Unsurprisingly, 74% of failed intubations were rescued with video laryngoscopes in our review. Moreover, video laryngoscopy was the primary choice in cases of anticipated difficult intubations. But a meta-analysis demonstrated that while video laryngoscopy improved glottis visualization in children, this device prolonged the intubation time and increased the incidence of failed intubations [10]. A possible explanation may lie in the pediatric endotracheal tube does not always pass straight through the vocal cords when guided by the video laryngoscopy blade. Hence, video laryngoscopy are not recommended in children who may not tolerate long periods of apnea.

In our review, the practice of fiberoptic-assisted tracheal intubation was applied with a low frequency(4.2%, 13/307). The introduction of the video laryngoscopy might weaken the education of trainees for fiberoptic-assisted tracheal intubation, which is a skill that takes time to master and maintain [9]. Regardless of which airway-visualizing devices is chosen, the primary goal of maintaining the pediatric airway is to ensure oxygenation and ventilation [11]. Both expected and unexpected difficult intubation mean oxygenation impairment, and neither the video laryngoscopy nor the fiberoptic bronchoscope are the solution.

For children who were bilateral HFM, airway management should be approached cautiously. The small mandible and severely limited submandibular space restrict vocal cords exposure during direct laryngoscopy. In the study of Nagorzian et al. [12], the incidence of difficult intubation in bilateral HFM cases was almost threefold higher than that in cases of unilateral HFM. Our review included 4 patients with bilateral involved HFM, 3 patients required airway-visualizing equipment for a successful intubation and 1 patient underwent a blind intubation with a direct laryngoscope. However, our review did not compare the influences of unilateral and bilateral HFM on laryngoscopic view for absence of laryngoscopic view assessment for bilateral HFM absence. Anesthesiologists prefered airway-visualizing equipment for intubation in bilateral HFM impling high risk of difficult intubation by airway assesement. Although airway-visualizing equipment could increase success rate of intubation, it still need to note that the possibility of difficult mask ventilation in bilateral HFM children. Hence, it is rational and safe to intubate under spontaneous breathing.

In this study, the IIG, TMD, and FPM grades were independent predictors of DLV in children with HFM. MMP, the most commonly used airway assessment test for adults, was unable to predict DLV. A possible explanation for this finding is that children with HFM are likely to have a smaller submandibular space than adults, and the MMP does not accurately predict poor visualization of the glottis during direct laryngoscopy. No predictive test can have 100% sensitivity and specificity for DLV. However, various preoperative tests could reduce the incidence of unanticipated failures in visualizing laryngeal structures and the number of potentially unnecessary interventions required when a difficult airway is not anticipated. In our study, the sensitivity, specificity, and AUC for the combination of TMD, IIG, and FPM were 94.12, 88.57%, and 0.782, respectively. The use of this combination is the best method to predict DLV in HFM children.

Mandibular distraction osteogenesis allows for increasing the vertical length of the mandible, increasing the bone stock, improving soft tissue asymmetry and reducing relapse [13], thereby altering the laryngoscopic view during tracheal intubation. Latency period was allowed for 4 to 7 days before distraction began. The distraction device was initiated at a rate of 1 mm/day. Generally, the overall distraction distances range from 20 to 40 mm, according to the preoperative design. Afterward, the distractor was left in place for 4 to 13 months for consolidation. The distraction device is continuously used until it is removed during the second-stage operation [1]. Our results show that 43% children (26/60) improved laryngeal exposure and decrease intubation difficulty under direct laryngoscopy during the second-stage operation. These results are consistent with those of previous studies that mandibular distraction osteogenesis could improve the laryngeal view, increase the airway volume and mandibular volume [14–16]. However, some complications can occur after surgical correction, such as infection and ankylosis of the temporomandibular joint, which can cause intubation to be more difficult. Hence, for patients who require a procedure after an initial mandibular distraction, the airway should be approached more cautiously.

Our study has some limitations. This is a retrospective study, which has inherent limitations. No prospective standardization in airway management was performed or recorded by the anesthetists. The completeness and accuracy of the data were somewhat limited in the anesthesia records. Future research in airway management for HFM patients should be prospective with standardized protocols for airway management and utilize a survey form. Finally, we do not have detailed information on the airway complications that occurred during anesthesia or in the postanesthesia care unit (PACU). Further research might shed more light on this problem.

## Conclusion

Children with HFM have a higher incidence of DLV. Airway-visualizing equipment increases the first pass success rates of intubation compared with direct laryngoscopy, facilitates intubation in children with DLV. A prior mandibular distraction could improve laryngeal views, decrease the degree of intubation difficulty at the second stage of the operation. However, for all patients with HFM, alternative airway equipment should be prepared.

## Data Availability

The data set used/analysed during the current study are available from the corresponding author on reasonable request.
